# Higher correlation between neutralizing antibodies and surrogate neutralizing or binding antibodies in COVID-19 patients than vaccine recipients

**DOI:** 10.1371/journal.pone.0298033

**Published:** 2024-04-16

**Authors:** Hatairat Lerdsamran, Ratikorn Anusorntanawat, Kantima Sangsiriwut, Suteema Sawadpongpan, Jarunee Prasertsopon, Nattakarn Thinpan, Poj Intalapaporn, Ranida Techasuwanna, Pilailuk Okada, Pilaipan Puthavathana

**Affiliations:** 1 Center for Research Innovation and Biomedical Informatics, Faculty of Medical Technology, Mahidol University, Nakhon Pathom, Thailand; 2 Chaophraya Yommarat Hospital, Office of the Permanent Secretary, Ministry of Public Health, Suphanburi, Thailand; 3 Department of Preventive and Social Medicine, Faculty of Medicine Siriraj Hospital, Mahidol University, Bangkok, Thailand; 4 Department of Medical Services, Rajavithi Hospital, Ministry of Public Health, Bangkok, Thailand; 5 Department of Disease Control, Ministry of Public Health, Nonthaburi, Thailand; 6 Department of Medical Science, Ministry of Public Health, Nonthaburi, Thailand; Qatar University, QATAR

## Abstract

This study determined the seropositive rates and levels of antibodies to severe acute respiratory syndrome coronavirus-2 in 50 patients and 108 vaccinees using microneutralization test (MNT), surrogate virus neutralization test (sVNT), chemiluminescent microparticle immunoassay (CMIA), and electrochemiluminescence immunoassay (ECLIA). MNT, as the reference method, employed living clade S and Delta viruses to measure neutralizing (NT) antibodies, while sVNT employed wild type strain and Delta receptor-binding domains (RBD) as the test antigens to measure sVNT antibodies. CMIA and ECLIA employed only one version of RBD to measure the binding antibodies. Our study performed *S* gene sequencing of the test virus to exclude undesired mutants that might lead to changes in antibody levels in MNT assay. We showed that spike protein amino acid sequences of our Delta virus contained 13 amino acid changes, with 3 related to the reduced neutralization. The MNT assay showed a significant reduction in seropositive rates and antibody levels in the patients’ sera when the Delta variant replaced clade S as the test virus. In contrast, the seropositive rates determined by sVNT assay using wild type strain RBD and Delta RBD were non-significantly different, suggesting that sVNT assay could not identify the difference between the antigenicity of wild type RBD and Delta RBD. Furthermore, the correlation between the levels of NT and sVNT antibodies was moderate with the patients’ sera but modest with the post-vaccination sera. The seropositive rates in the patients, as determined by CMIA or ECLIA, were not different from the MNT assay using clade S, but not Delta, as the test virus. In all analyses, the correlations between the antibody levels measured by MNT and the other 3 assays were modest to moderate, with the *r*-values of 0.3500–0.7882.

## Introduction

Immunocompetent individuals usually elicit the humoral and cellular immunity specific to severe acute respiratory syndrome coronavirus-2 (SARS-CoV-2) after natural infections or vaccinations [1, 2]. Some antibody molecules known as the “functional antibodies,” the so-called “neutralizing (NT) antibodies,” are the critical elements of humoral immunity through their roles in the protection against the infection or disease, or at least reduce the disease severity [3, 4]. On the other hand, the “non-neutralizing or binding antibodies” recognize the antigenic epitopes and play a role in the defense mechanism through antibody-dependent cell-mediated cytotoxicity, complement-dependent cell lysis, and opsonization [4]. NT antibodies bind to the receptor-binding domain (RBD) on the virus particles and prevent them from attaching to the cell receptors, i.e., blocking the viral entry. In addition, NT antibodies inhibit viral uncoating in the endosomes in case the virus particles enter target cells through the mechanism of receptor-mediated endocytosis [4].

Measurement of NT antibody levels is essential for evaluating the vaccine effectiveness and the therapeutic monoclonal antibodies used to treat coronavirus disease-19 (COVID-19). NT antibodies are highly strain-specific, so the variant strains that emerged over time could escape or limit the neutralizing activities of convalescent sera, post-vaccination sera, or therapeutic monoclonal antibodies [5–7]. Furthermore, conventional neutralization-based-assays are not easy to accomplish due to the requirement of technical skill and biosafety laboratory level 3 facilities. The assays also require many reagents, time, and tedious work on the cell and virus cultivation. Most of all, laboratorians are at risk of laboratory-acquired infection when working with SARS-CoV-2. Even though the antibody assays were employed to evaluate vaccine effectiveness and predict protective immunity [3, 8, 9], the determination of correlation between the levels of antibodies measured by conventional neutralization and other assays is limited.

In this study, we conducted a cytopathic effect (CPE) based-microneutralization test (MNT) to measure SARS-CoV-2 NT antibody titers in sera collected from COVID-19 patients and vaccinees after receiving a booster dose of COVID-19 vaccine. Furthermore, we correlated the levels of NT antibodies with the levels of surrogate virus neutralizing (sVNT) antibodies measured by sVNT (GenScript) assay and with the binding antibodies measured by CMIA (Abbott) and ECLIA (Roche) auto analyzers. Our MNT assay employed the SARS-CoV-2 clade S and the Delta (B.1.617.2) variant strain as the test viruses, the sVNT assay employed RBD derived from the wild type and Delta strains as the test antigens, while CMIA and ECLIA employed only one version of the test antigens.

## Materials and methods

### Vaccines and vaccine administration

The CoronaVac, an inactivated vaccine manufactured by Sinovac Life Sciences Co. Ltd., Beijing, P.R. China, containing 3 μg/dose, was administered intramuscularly. ChAdOx1-S or COVID-19 Vaccine AstraZeneca, a chimpanzee adenovirus-based-vector vaccine harboring complete *S* gene insertion and contained 5×10^10^ virus particles/dose employed intramuscularly injection [10].

### Subjects and serum sample collection

Archival serum specimens of 50 patients who got SARS-CoV-2 infection between February 2020 and June 2020, the first epidemic wave in Thailand (January to 14 December 2020, according to the Department of Disease Control, the Ministry of Public Health) [11]. These patients were laboratory-diagnosed by real-time reverse transcription polymerase chain reaction (RT-PCR). Unfortunately, we had no information about their disease severity. The time of blood sample collection was between 2 weeks to 2 months after disease symptoms. Each serum sample was labeled with a patient ID code and collection date, which we could not identify individual patients. The Central Institution Review Board (CIRB), Mahidol University approved using these patients’ sera under the protocol MU-COVID2020.001/2503. The CIRB waived the requirement for informed consent because the sera were the leftover samples from a clinical laboratory investigation.

Under the CIRB protocol MU-COVID2020.001/2503, we also received approval to collect blood samples from healthy participants for a sero-surveillance study. Later, those participants received two doses of the CoronaVac vaccine at 3-week intervals, followed by a booster dose of the ChAdOx1-S vaccine 2–3 months after that (2 CoronaVac + 1 ChAdOx1-S). The study included 108 vaccinees whose blood samples were collected one month after the booster vaccination under the CIRB approval for protocol number MU-CIRB 2021/202.2204. The recruitment period was from March to November 2021. Participants received an explanation and signed the informed consent form for participation. The demographic data of participants in this study is shown in Table 1. We used the serum samples collected for the sero-surveillance study as the pre-vaccination sera or baseline control for comparison with the post-booster vaccination sera. The baseline serum samples were negative for SARS-CoV-2 anti-N antibody and had neutralizing antibody titer <10. Blood specimens were labeled with study ID numbers. As most of the participants in the vaccine group are health workers, they were monitored weekly free of SARS-CoV-2 infection by real-time RT-PCR or antigen detection throughout the epidemics, and none of them were infected. We concluded that none of them got COVID-19 infection during the study period.

**Table 1:** Demographic data of participants in this study.

Groups	Number of participants in each group	Number of participants with available age data	Age, years		Gender, n		Blood collection date
			Mean	Range	Males	Females	
COVID-19 patients	50	34	40.1	17–89	27	23	10 Mar– 7 Aug 2020
COVID-19 vaccinees	108	108	41.3	23–70	27	81	29 Mar– 18 May 2021 (Pre-vaccination/ baseline sera)
							19 Aug—30 Nov 2021 (Sera collected after booster vaccination)

### The study viruses

The test viruses used in MNT assays were SARS-CoV-2 clade S (designated hCoV-19/TH/MUMT-3/2020) and Delta or B.1.617.2 variant (designated hCoV-19/TH/MUMT-53/2021). These two viruses had been isolated and propagated in Vero cells before nucleotide sequencing and using in MNT assay. We deposited their sequences in the GISAID database with the accession numbers EPI_ISL_493137 and EPI_ISL_14780257 for hCoV-19/TH/MUMT-3/2020 clade S and hCoV-19/TH/MUMT-53/2021 Delta variant, respectively.

### Microneutralization test (MNT)

We conducted the CPE-based MNT assay for NT antibody titers according to the protocol described in our previous studies [12, 13]. Briefly, the test sera were heat-inactivated at 56°C for 30 minutes and serially two-fold-diluted from the initial dilution of 1:10 to 1:1280. Next, a volume of 60 μl of each serum dilution was mixed with a volume of 60 μl of the test virus at a concentration of 200 TCID_50_ (50% tissue culture infectious dose)/100 μl to make the final virus concentration of 100 TCID_50_/reaction well in micro-culture plates. The reaction plates were incubated at 37°C for one hour before 100 μl of serum-virus mixture was transferred onto a well of Vero cell monolayer in duplicate. We examined the reaction plates for CPE daily before reading the result after incubation for 3 days at 37°C. The NT antibody titer is the reciprocal of the highest serum dilution that inhibits higher than 50% CPE in the wells inoculated with the serum-virus mixture compared to the control wells comprising the virus controls and the uninfected cell monolayer controls.

### Surrogate virus neutralization test (sVNT)

The cPass^TM^ SARS-CoV-2 NT antibody detection kit (Nanjing GenScript Biotech Co., Ltd., Nanjing, China) is a competitive ELISA for the detection of sVNT antibodies against SARS-CoV-2. According to the kit instruction, 60 μl of the test serum at dilution 1:10 in a single well or the positive and negative controls at dilution 1:10 in duplicate wells were incubated at 37°C for 30 minutes with 60 μl of horseradish peroxidase-conjugated-RBD (HRP-wild type RBD or HRP-Delta RBD) at dilution of 1:1000. Then, a 100 μl volume of the serum-RBD mixture was transferred to a reaction well of the 96 well-microtiter plates pre-coated with human angiotensin-converting enzyme-2 (hACE2) and further incubated at 37°C for 15 minutes. Next, the tetramethylbenzidine (TMB) chromogenic substrate solution was added to the reaction wells, followed by incubation for 15 minutes at room temperature in the dark before stopping the reaction. The plate was read for optical density (OD) using a microplate spectrophotometer at the wavelength of 450 nm. The percentages of signal inhibition were determined based on the kit instruction [% inhibition = 1- (OD value of sample/OD value of negative control) × 100]. The signal inhibition ≥30.00% was considered positive for sVNT antibodies against the test RBD. The sVNT assay employed two kinds of RBD derived from wild type strain and Delta variant of SARS-CoV-2.

### Chemiluminescent microparticle immunoassay (CMIA)

SARS-CoV-2 IgG II Quant assay (Abbott Ireland Diagnostics Division, Ireland) is a chemiluminescent microparticle immunoassay (CMIA) for the qualitative and quantitative determination of IgG antibodies against RBD using an ARCHITECT i1000SR system autoanalyzer. The SARS-CoV-2 IgG in the test serum bound to the RBD-coated-paramagnetic microparticles and subsequently to acridinium-conjugated-mouse anti-human IgG. Measurement of the chemiluminescence signals emitted from the antigen-antibody complexes yielded the relative light units (RLU), which directly correlated with the amount of SARS-CoV-2 IgG in the test serum. The operation system displayed the test result in terms of AU/ml, which after multiplying with 0.142, resulted in the WHO International Standard units (BAU/ml). The manufacturer defines a test serum with ≥50.00 AU/ml or ≥7.10 BAU/ml as positive for SARS-CoV-2 IgG antibodies.

### Electrochemiluminescence immunoassay (ECLIA)

The ECLIA used in this study was the Elecsys^®^ anti-SARS-CoV-2 S assay (Roche Diagnostics GmbH, Germany) for *in vitro* quantifying the antibodies (including IgG) to SARS-CoV-2 S protein RBD using a Cobas e 411 autoanalyzer. The biotinylated-RBD and the ruthenium labeled-RBD bound specific antibodies in the test serum to form double-antigen sandwich complexes. With streptavidin-coated microparticles added into the reaction, biotin on the complexes bound to streptavidin on the microparticles. The reaction emitted chemiluminescent signals that were measured by a photomultiplier. The values of ≥0.80 U/ml were the cut-off for positive anti-SARS-CoV-2-S antibodies. The optimal range of quantitation was 0.40 to 250.00 U/ml or up to 25000.00 U/mL for 100-fold diluted samples.

### Statistical analysis

We employed GraphPad Prism 9 version 9.5.1 for calculating mean, geometric mean titer (GMT), and Spearman correlation (*r*-value). Moreover, we used PASW statistics 18 version 18.0.0 for calculating the sensitivity, specificity, and Wilcoxon Signed Ranks test (*p*-value).

## Results

### Nucleotide sequences encoded S proteins of the test viruses

We aligned S amino acid sequences of our SARS-CoV-2-clade S (hCoV-19/TH/MUMT-3/2020) and Delta virus (hCoV-19/TH/MUMT-53/2021) against the NC_045512.2 Wuhan-Hu-1 ancestral virus (S1 Fig). Throughout the length of 1273 amino acids of S protein, we found 1 amino acid change (A829T) in clade S virus and 13 changes (T19R, G142D, E156G, F157 deletion, R158 deletion, S247R, L452R, T478K, D614G, P681R, D950N, K986E, and Q1002H) in Delta virus compared with the Wuhan-Hu-1 reference strain. Confining to the RBD regions at amino acid positions 319–541, the result showed no amino acid change in our clade S virus and 2 changes in the receptor binding motif (L452R and T478K) in the Delta variant.

### MNT and sVNT assays in COVID-19 patients

**Fig 1:**
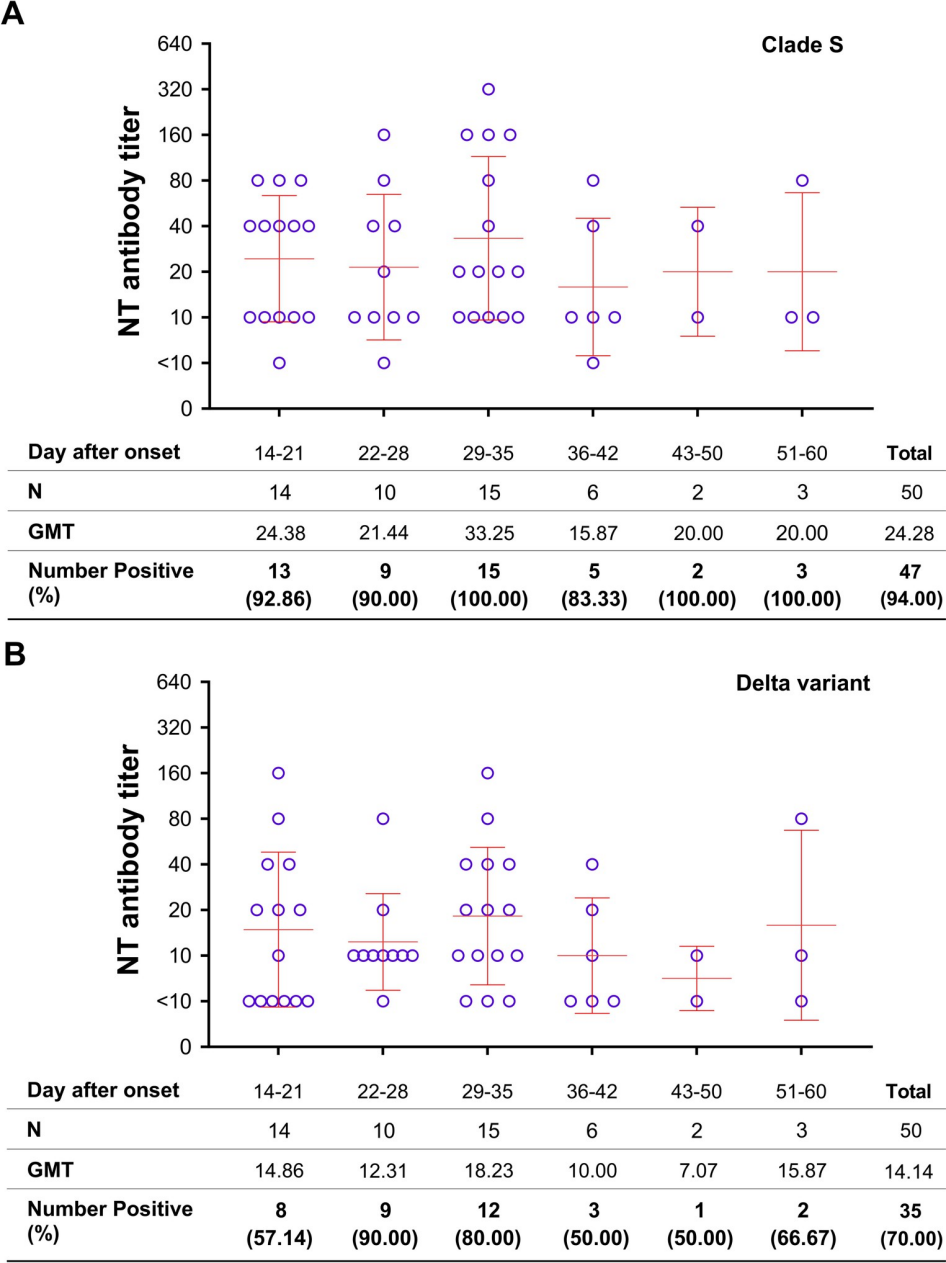
MNT assay for anti-SARS-CoV-2 NT antibodies in COVID-19 patients. A) Tested with clade S virus; B) Tested with Delta virus. (Error bars represent GMT ± SD).

We investigated single serum samples from 50 COVID-19 patients by CPE based-MNT assay to determine the anti-SARS-CoV-2 NT antibodies using clade S and Delta as the test viruses. The result showed a significantly higher seropositive rate against clade S virus when compared with the Delta virus, i.e., 94.00% v.s. 70.00% (Wilcoxon Signed Ranks test; *p* = 0.001) (Table 2). All COVID-19 patients developed the antibody response to clade S virus at 4–5 weeks, and NT antibodies lasted for two months after the onset of symptoms as investigated that far (Fig 1A), while 90.00% of them developed the antibody response to the Delta variant at 3–4 weeks before gradual declining (Fig 1B). The NT antibody titers among the patients were at a range of <10 to 320 with GMT of 24.28 with the clade S virus (Fig 1A) and a range of <10 to 160 with GMT of 14.14 with Delta virus (Fig 1B). The levels of NT antibody titers to clades S virus were also significantly higher than to Delta virus (Wilcoxon Signed Ranks test; *p* = 0.001).

**Table 2:** Number of seropositive cases as determined by various methods.

Subjects	N	Number positive (%) by					
		MNT^a^		sVNT^b^		CMIA^c^	ECLIA^d^
		Clade S	Delta	Wild type	Delta		
**COVID**-**19 patients**	50	47 (94.00%)	35 ***(70.00%)	48 (96.00%)	46 ^NS^(92.00%)	49 (98.00%)	49 (98.00%)
**COVID**-**19 vaccinees**	108	108 (100.00%)	107 ^NS^(99.07%)	108 (100.00%)	108 ^NS^(100.00%)	108 (100.00%)	108 (100.00%)

a. MNT positive: antibody titer ≥10

b. sVNT positive: % inhibition ≥30.00%

c. CMIA positive: ≥7.10 BAU/ml

d. ECLIA positive: ≥0.80 U/ml

Note

*** = significant difference (*p*-value = 0.001), NS = non-significant difference (Wilcoxon Signed Ranks test)

**Fig 2:**
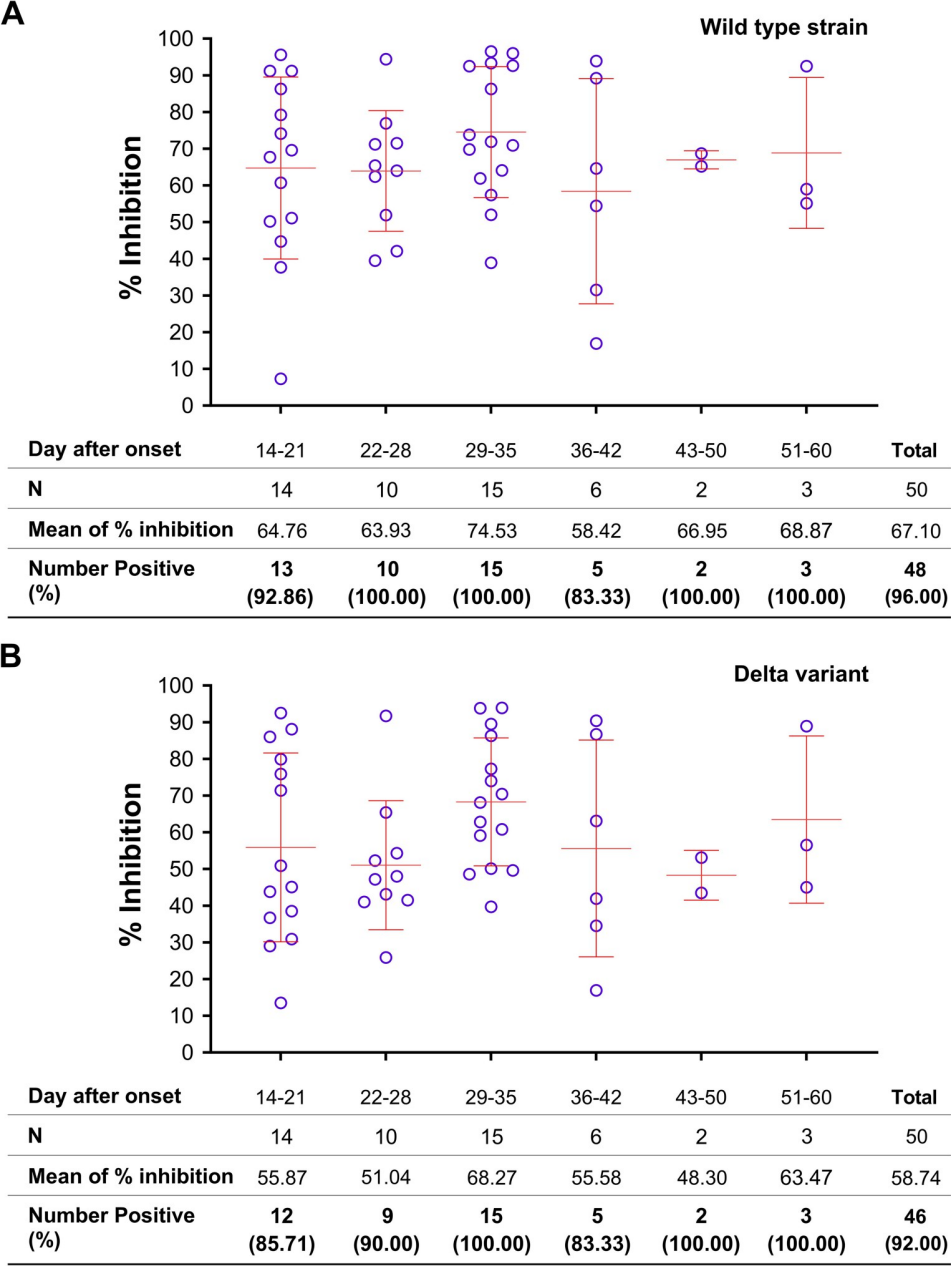
sVNT assay for sVNT antibodies to SARS-CoV-2 in COVID-19 patients. A) Tested with wild type RBD; B) Tested with Delta RBD. (Error bars represent GMT ± SD).

The sVNT assay used wild type RBD and Delta RBD as the test antigens for anti-SARS-CoV-2 sVNT antibodies. The investigation in COVID-19 patients showed the seropositive rates of 96.00% with wild type RBD and 92.00% with Delta RBD, which were statistically non-significant different (Wilcoxon Signed Ranks test; *p* = 0.157) (Table 2). All COVID-19 patients developed sVNT antibodies to wild type RBD at 3 weeks after the onset of symptoms and persisted for at least 2 months, with the percentages of signal inhibition varying from 7.30 to 96.47% (mean = 67.10%) (Fig 2A). Similarly, all patients developed sVNT antibodies to Delta RBD 4–5 weeks after disease onset, with the percentages of signal inhibition varying from 13.50 to 93.90% (mean = 58.74%) (Fig 2B). The levels of sVNT antibodies to both wild type and Delta RBDs were significantly different (Wilcoxon Signed Ranks test; *p* = 0.000) (Fig 2A and 2B).

**Fig 3:**
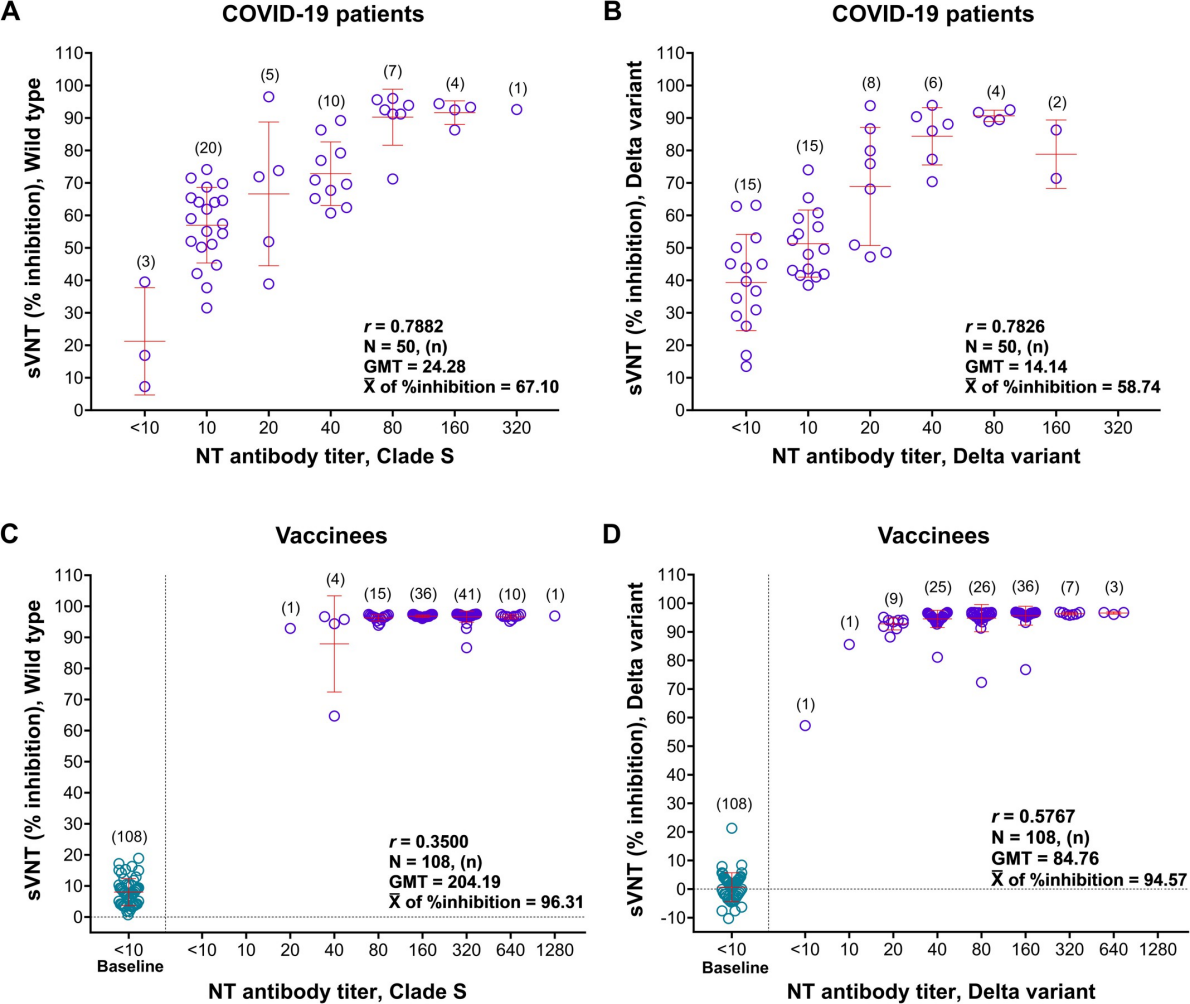
Correlation between the levels of anti-SARS-CoV-2 NT antibodies measured by MNT and sVNT assays. A) COVID-19 patients’ sera tested with clade S/wild type; B) COVID-19 patients’ sera tested with Delta variant; C) vaccinees’ sera tested with clade S/wild type; and D) vaccinees’ sera tested with Delta variant.

This study also compared the SARS-CoV-2 seropositive rates in COVID-19 patients using MNT against sVNT assay and obtained the values of 94.00% v.s. 96.00% for clade S/wild type, and 70.00% v.s. 92.00% for Delta variant, respectively. The differences in seropositive rates were statistically non-significant with clade S/wild type (Wilcoxon Signed Ranks test; *p* = 0.3170), but highly significant with the Delta variant (Wilcoxon Signed Ranks test; *p* = 0.001) (Table 2). Nevertheless, the analysis showed a moderate correlation between the levels of NT and sVNT antibodies, with the *r-*values of 0.7882 for clade S/wild type (Fig 3A) and 0.7826 for the Delta variant (Fig 3B).

### MNT and sVNT assays in post-vaccination sera

Of 108 post-vaccination sera, the MNT assay showed the seropositive rates of 100.00% with clade S virus and 99.07% with Delta virus, which were statistically non-significantly different (Wilcoxon Signed Ranks test, *p* = 0.317) (Table 2). Moreover, the MNT assay showed the NT antibody titers at a range of 20 to 1280 (GMT = 204.19) with clade S virus (Fig 3C) and <10 to 640 (GMT = 84.76) with Delta virus (Fig 3D). The statistical analyses on the levels of NT antibody titers in post-vaccination sera, as measured by these two viruses using MNT assay, were significantly different (Wilcoxon Signed Ranks test, *p =* 0.000).

The investigation in post-vaccination sera using sVNT assay showed 100.00% seropositive rates with both wild type and Delta RBDs (Table 2). The percentages of signal inhibition varied from 64.68 to 97.51% (mean = 96.31%) with wild type RBD antigen and from 57.16 to 96.95% (mean = 94.57%) with Delta RBD (Fig 3C and 3D). This signal inhibition with both RBD antigens differed significantly (Wilcoxon Signed Ranks test, *p* = 0.000). Nevertheless, the correlation between the levels of NT antibodies obtained from MNT assay and the percentages of signal inhibition obtained from sVNT assay was poor, with the *r*-values of 0.3500 for clade S/wild type (Fig 3C), and 0.5767 for Delta variant (Fig 3D).

### CMIA for anti-SARS-CoV-2 binding antibodies

**Fig 4:**
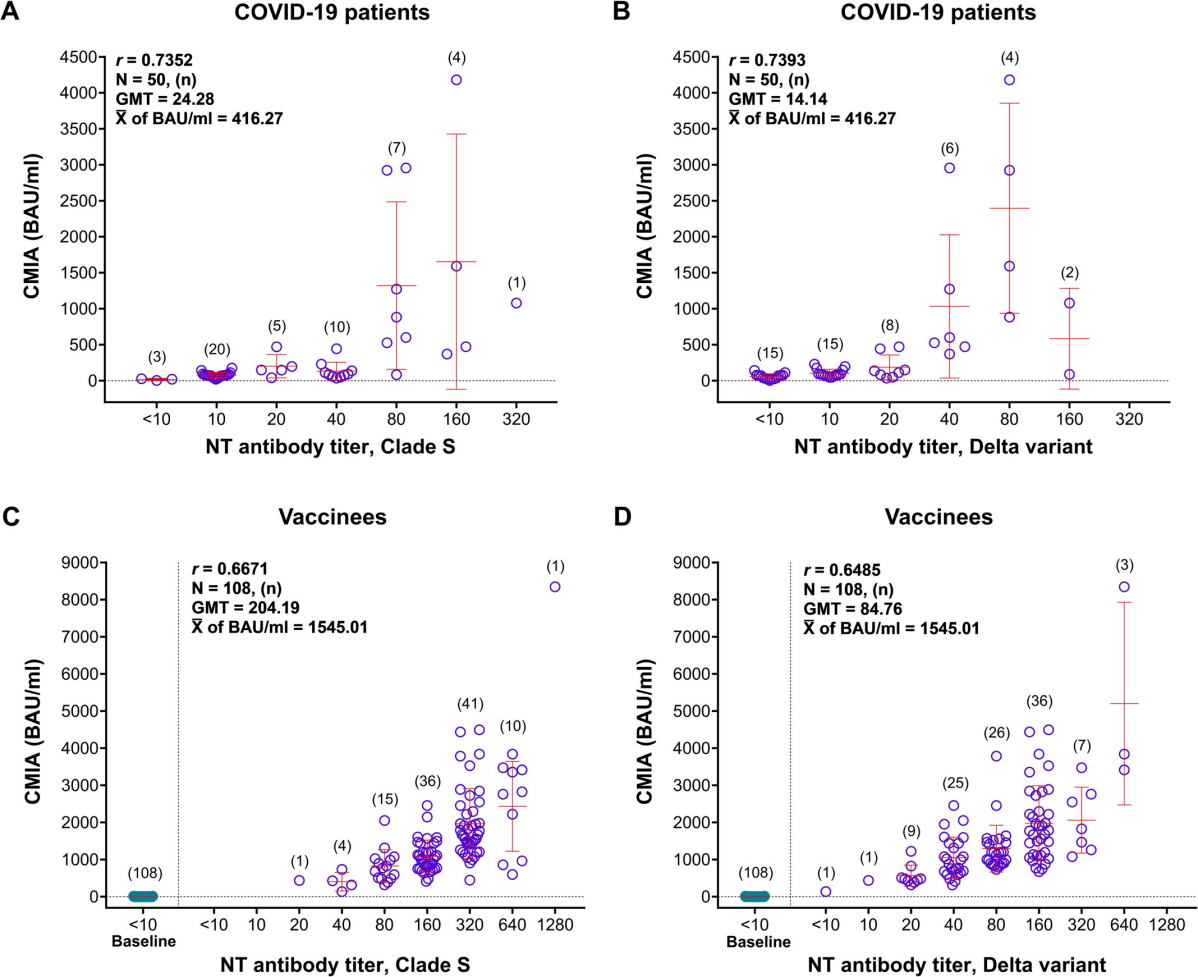
Correlation between the binding antibodies measured by CMIA and NT antibodies measured by MNT assay. A) COVID-19 patients’ sera tested with clade S virus; B) COVID-19 patients’ sera tested with Delta variant; C) vaccinees’ sera tested with clade S virus; and D) vaccinees’ sera tested with Delta variant.

CMIA SARS-CoV-2 IgG II Quant assay employed only one version of RBD as the test antigen over time. The assay yielded 98.00% seropositive rates in the patients and 100.00% in the vaccinees (Table 2). In COVID-19 patients, the assay showed the binding antibody levels at 2.39 to 4180.11 BAU/ml (mean = 416.27, and median = 87.35 BAU/ml). The levels of BAU moderately correlated with the level of NT antibody titers determined by MNT assay, with the *r*-values of 0.7352 and 0.7393 for clade S and Delta virus, respectively (Fig 4A and 4B).

Moreover, the vaccinees’ sera contained the SARS-CoV-2 IgG antibodies at 136.45 to 8342.76 BAU/ml (mean = 1545.01, and median = 1199.77 BAU/ml). The correlation between MNT antibody titers and CMIA SARS-CoV-2 IgG concentration was also moderate, with the *r*-values of 0.6671 for clade S virus and 0.6485 for Delta virus (Fig 4C and 4D).

### ECLIA for anti-SARS-CoV-2 binding antibodies

**Fig 5:**
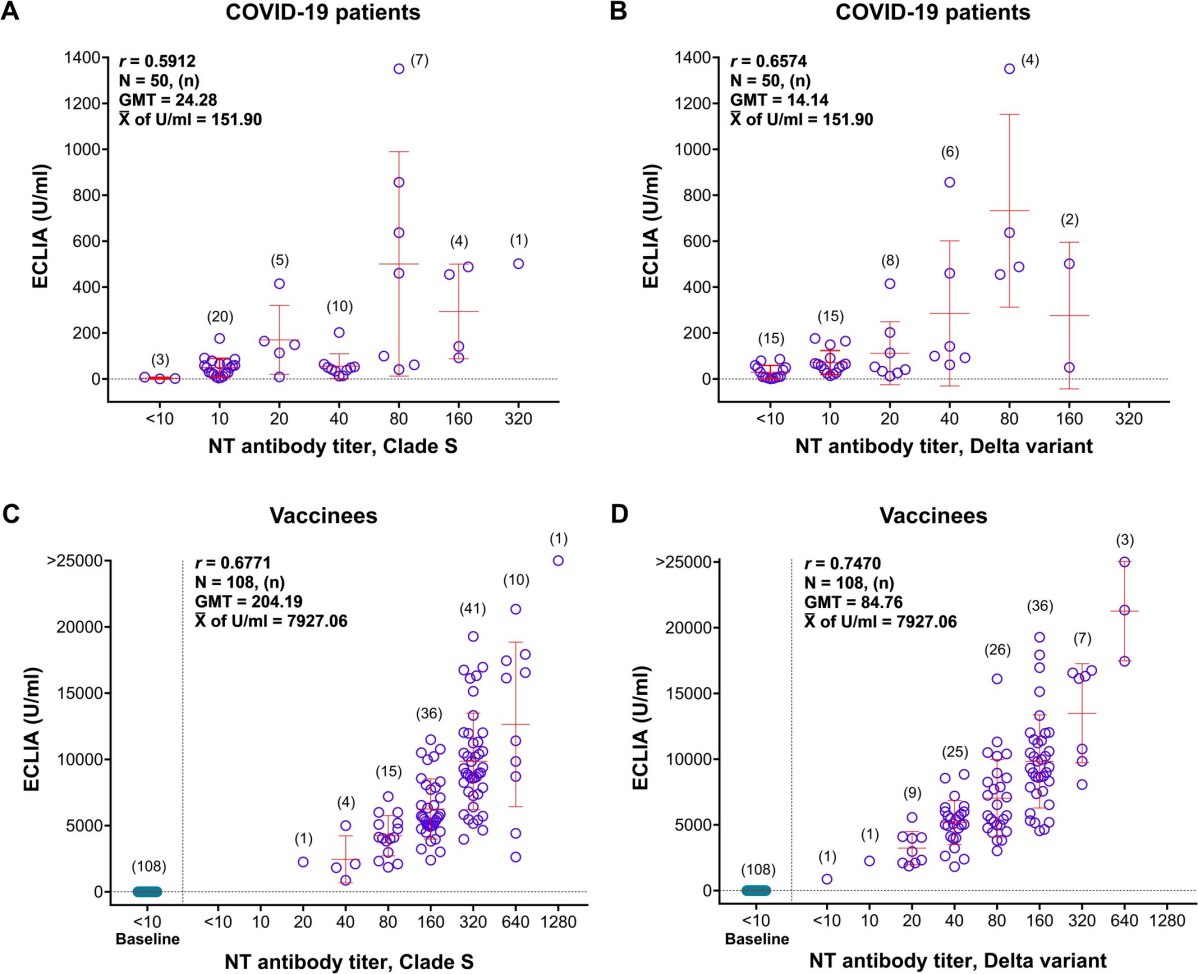
Correlation between the binding antibodies measured by ECLIA and NT antibodies measured by MNT assay. A) COVID-19 patients’ sera tested with clade S virus; B) COVID-19 patients’ sera tested with Delta variant; C) vaccinees’ sera tested with clade S virus; and D) vaccinees’ sera tested with Delta variant.

ECLIA also employed only one version of RBD antigen over time to measure anti-SARS CoV-2 antibodies. The assay yielded 98.00% seropositive rates in the patients and 100.00% in the vaccinees (Table 2). In COVID-19 patients, the assay showed the binding antibody levels at <0.40 to 1351.00 U/ml (mean = 151.90, and median = 57.73 U/ml). In the patients, the antibody levels determined by MNT and ECLIA moderately correlated with the *r*-values of 0.5912 and 0.6574 when the MNT assay employed clade S and Delta as the test viruses, respectively (Fig 5A and 5B).

In the post-vaccination sera, the level of anti-SARS CoV-2 binding antibodies determined by ECLIA was 872.00 to >25000.00 U/ml (mean = 7927.06, and median = 6821.00 U/ml). The ECLIA moderately correlated with the MNT assay, with the *r*-values of 0.6771 and 0.7470 when the MNT assays employed clade S and Delta as the test virus, respectively (Fig 5C and 5D).

## Discussion

The present study included 4 assays to measure anti-SARS-CoV-2 antibodies. The MNT assay measured NT antibodies, the sVNT assay measured sVNT antibodies, and CMIA and ECLIA measured non-NT or binding antibodies. The MNT assay focused on live viruses, while the other three assays targeted the RBD test antigens. The protective antibodies mainly targeted epitopes on the S protein.

SARS-CoV-2 S protein is 1,273 amino acids long. The cleavage of S protein at the furin cleavage site (amino acid positions 680–685) yielded the S1 and S2 domains. RBD, at a length of 223 amino acids, is located at positions 319–541 as part of the S1 domain. The RBD binds directly to the hACE2 cell receptor and mediates the viral entry [14, 15]. Hence, RBD plays a significant role as the principle-neutralizing domain of SARS-CoV-2. Unfortunately, RBD is the most variable region and has undergone genetic change over time [16]. Moreover, collective findings showed that neutralizing epitopes are not only present in RBD but also in the other regions of S protein, i.e., the N-terminal domain (NTD) [17–20] and the S2 domain [21, 22]. These findings showed that our conventional MNT assay using live virus should provide the most accurate result because the NT antibodies recognized NT epitopes that lined along the S protein. In contrast, the other 3 assays measured the antibody that targeted only the epitopes on RBD.

This study recruited serum samples from the patients who got SARS-CoV-2 infection between February 2020 and June 2020 during the first epidemic wave, which peaked in March. The major circulating viruses of the first wave belonged to clade O and followed by clade S, according to the data recorded in GISAID. Nevertheless, viruses of various clades (S, O, L, G, and V) were identified, but clade S (PANGO lineages A or A.6) was the predominant virus at the early phase of the epidemic [23–25]. Unfortunately, the causative viruses of our patients were not genetically characterized. We chose clade S virus as a test virus in MNT assay to go along with the virus clade used in vaccines of various manufacturers, and we also chose a Delta variant as another test virus to determine the change in antibody levels according to the viral genetic change.

To our experience on the isolation and propagation of SARS-CoV-2 in Vero cells, amino acid changes found in S protein were frequent, compared to the original sequences discovered in direct specimens. The most serious was the occurrence of 3 adjacent amino acid changes just after 3 subpassages of cultivation. MNT assay using the undesired mutants as the test virus might yield an inaccurate result of the antibody measurement. This concern has never been raised in other previous studies. Therefore, we sequenced the S proteins and chose the test viruses before conducting the MNT experiments. Using the S amino acid sequence of the ancestral strain as the reference, our alignment revealed 13 amino acid changes in Delta, compared to 1 change in clade S. Of these 13 changes in Delta, at least G142D in NTD [19], L452R and T478K substitutions in RBD [5, 26], contributed to the resistance of Delta variant to neutralization as reported previously. We cannot exclude that the other substitution positions may also disrupt the neutralizing activities. Our report could be helpful for further study on the evaluation of neutralizing antibody activity.

The present study in COVID-19 patients showed a significant reduction in seropositive rates and the NT antibody levels when the Delta variant replaced clade S as the test virus in MNT assay. In contrast, the difference in seropositive rates determined by sVNT assay using wild type RBD and Delta RBD was non-significant, even though the difference was significant with the levels of sVNT antibodies. This study implied that sVNT could not sufficiently characterize the antigenic difference between wild type RBD and Delta RBD. Moreover, the antibody levels in the patients’ sera, as determined by MNT assay and sVNT assay using clade S/wild type and Delta, moderately correlated with the *r*-values of 0.7882 and 0.7826, respectively.

Due to robust antibody response after vaccination, almost all study sera were seropositive with MNT or sVNT assay, regardless of clade S or Delta virus testing. The superiority of the MNT assay over the sVNT assay relied on the breadth of NT epitopes located along the S protein. In contrast, the sVNT assay only detected the sVNT antibodies against neutralizing epitopes in the RBD. Correlation between the levels of antibodies obtained from MNT and sVNT assays in post-vaccination sera was poor or modest, as shown by the *r*-values of 0.3500 and 0.5767 with clade S/wild type and Delta viruses, respectively. We could not explain why MNT and sVNT assays correlated more with the patients’ sera (*r*-values of 0.7882 with clade S/wild type, and 0.7826 with Delta virus), than the post-vaccination sera. In contrast, the other group of investigators reported a higher *r*-value of correlation between neutralizing antibodies and sVNT antibodies with post-vaccination sera [27].

This study also determined the correlation between NT antibodies and binding antibodies obtained from CMIA and ECLIA kits. Relying on the CMIA kit instruction, we multiplied the original arbitrary units (AU) with 0.142 to harmonize with the WHO international standard units. We expressed the result in terms of BAU/ml. On the other hand, the ECLIA kit instruction did not provide a calibration factor. Relying on the WHO NIBSC 20/136 standard pooled plasma, 1 original unit/ml by ECLIA was equal to 1.029 BAU/ml (multiplying factors of 1.029) [28, 29]. However, some investigators used the multiplying factors of 0.972 [30, 31]. Therefore, we expressed the binding antibody concentrations as the original units/ml. This study also showed that the binding antibody concentrations measured by CMIA and ECLIA reagents better correlated with neutralizing antibodies than the sVNT antibodies in all comparisons.

Conclusively, the correlation among the levels of antibodies obtained from all 3 methods (sVNT, CMIA, and ECLIA) yielded the *r*-values of less than 0.8 (range 0.3500–0.7882) when analyzed against MNT assay, regardless of the sources of the test sera, the patients or the vaccine recipients. Moreover, our study showed that high antibody levels in post-vaccination sera are not appropriate for multiple test comparisons.

Our data was limited to anti-SARS-CoV-2 antibodies to clade S and Delta virus, while the XBB variant is circulating worldwide. Our result found that using a new variant as the test virus in MNT assay may reduce NT antibody titers. Therefore, the units of binding antibody concentration that extrapolated to an NT antibody titer should be changed accordingly. In other words, the *r*-values for the correlation between levels of NT antibodies and the binding antibody units are not standing still but should be adjusted along with the new virus.

## Supporting information

S1 Fig Alignment of S protein amino acid sequences of our clade S and Delta viruses with the Wuhan-Hu-1 ancestral virus. Throughout the entire S protein of 1273 amino acids in length, RBD spans the positions 319–541, and RB motif in a rectangular box spanning the positions 437–508. One amino acid change (A829T) is present in clade S virus, and 13 changes (T19R, G142D, E156G, F157 deletion, R158 deletion, S247R, L452R, T478K, D614G, P681R, D950N, K986E, and Q1002H) in Delta variant. These mutation positions are shown in vertical boxes. According to previous investigators [5, 19, 22], 3 amino acid substitutions (G142D, L452R, and T478K), as shown in blue boxes, correlate with the reduction in neutralizing antibody titers.
(PDF)
