# Supplementary material for: Higher correlation between neutralizing antibodies and surrogate neutralizing or binding antibodies in COVID-19 patients than vaccine recipients
Source: PLoS One. 2024 Apr 16;19(4):e0298033. doi: 10.1371/journal.pone.0298033 (PMC11020499; doi:10.1371/journal.pone.0298033)
Supplement: S1 Fig — Throughout the entire S protein of 1273 amino acids in length, RBD spans the positions 319–541, and RB motif in a rectangular box spanning the positions 437–508. One amino acid change (A829T) is present in clade S virus, and 13 changes (T19R, G142D, E156G, F157 deletion, R158 deletion, S247R, L452R, T478K, D614G, P681R, D950N, K986E, and Q1002H) in Delta variant. These mutation positions are shown in vertical boxes. According to previous investigators [5, 19, 22], 3 amino acid substitutions (G142D, L452R, and T478K), as shown in blue boxes, correlate with the reduction in neutralizing antibody titers. (PDF) [file pone.0298033.s001.pdf]

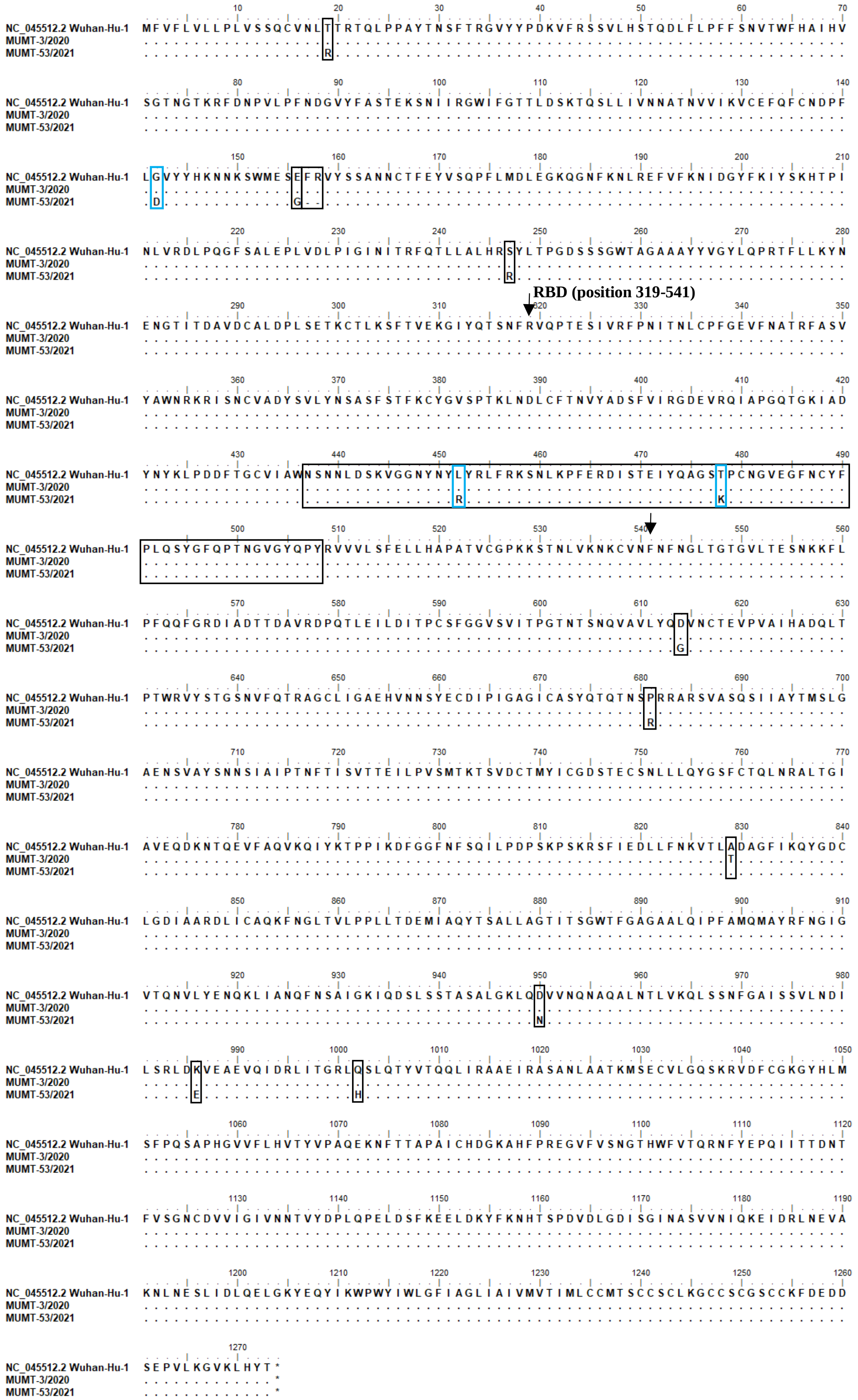
NC_045512.2 Wuhan-Hu-1
MUMT-3/2020
MUMT-53/2021
RBD (position 319-541)

S1 Fig. Alignment of S protein amino acid sequences of our clade S and Delta viruses with the Wuhan-Hu-1 ancestral virus. Throughout the entire S protein of 1273 amino acids in length, RBD spans the positions 319-541, and RB motif in a rectangular box spanning the positions 437-508. One amino acid change (A829T) is present in clade S virus, and 13 changes (T19R, G142D, E156G, F157 deletion, R158 deletion, S247R, L452R, T478K, D614G, P681R, D950N, K986E, and Q1002H) in Delta variant. These mutation positions are shown in vertical boxes. According to previous investigators [5, 19, 22], 3 amino acid substitutions (G142D, L452R, and T478K), as shown in blue boxes, correlate with the reduction in neutralizing antibody titers.
